# Investigating Arousal, Saccade Preparation, and Global Luminance Effects on Microsaccade Behavior

**DOI:** 10.3389/fnhum.2021.602835

**Published:** 2021-03-05

**Authors:** Jui-Tai Chen, Rachel Yep, Yu-Fan Hsu, Yih-Giun Cherng, Chin-An Wang

**Affiliations:** ^1^Department of Anesthesiology, Shuang Ho Hospital, Taipei Medical University, Taipei City, Taiwan; ^2^Department of Anesthesiology, School of Medicine, College of Medicine, Taipei Medical University, Taipei City, Taiwan; ^3^Centre for Neuroscience Studies, Queen’s University, Kingston, ON, Canada; ^4^Research Center of Brain and Consciousness, Shuang Ho Hospital, Taipei Medical University, Taipei City, Taiwan; ^5^Graduate Institute of Mind, Brain, and Consciousness, Taipei Medical University, Taipei City, Taiwan; ^6^Institute of Cognitive Neuroscience, College of Health Science and Technology, National Central University, Taoyuan City, Taiwan

**Keywords:** emotion, superior colliculus, pupil light and darkness reflex, emotional valence, fatigue-related arousal

## Abstract

Microsaccades, small saccadic eye movements occurring during fixation, have been suggested to be modulated by various sensory, cognitive, and affective processes relating to arousal. Although the modulation of fatigue-related arousal on microsaccade behavior has previously been characterized, the influence of other aspects of arousal, such as emotional arousal, is less understood. Moreover, microsaccades are modulated by cognitive processes (e.g., voluntary saccade preparation) that could also be linked to arousal. To investigate the influence of emotional arousal, saccade preparation, and global luminance levels on microsaccade behavior, emotional auditory stimuli were presented prior to the onset of a fixation cue whose color indicated to look either at the peripheral stimulus (pro-saccade) or in the opposite direction of the stimulus (anti-saccade). Microsaccade behavior was found to be significantly modulated by saccade preparation and global luminance level, but not emotional arousal. In the pro- and anti-saccade task, microsaccade rate was lower during anti-saccade preparation as compared to pro-saccade preparation, though microsaccade dynamics were comparable during both trial types. Our results reveal a differential role of arousal linked to emotion, fatigue, saccade preparation, and global luminance level on microsaccade behavior.

## Introduction

Even during periods of visual fixation, our eyes are not perfectly still. Instead, small fixational eye movements such as drift, tremor, and microsaccades are commonly observed. Over the past two decades, microsaccades have been studied extensively, given their modulation by various sensory and cognitive processes (Rolfs, 2009; Hafed, 2011; Martinez-Conde et al., 2013), their potential role as a biomarker for clinical investigation (Alexander et al., 2018; Yep et al., 2018), and their practical implications such as detecting visual fatigue during driving (Di Stasi et al., 2015). It is generally accepted that the superior colliculus (SC), a midbrain sensorimotor structure, is causally involved in the generation of both macrosaccades and microsaccades (Hafed, 2011; Hafed and Krauzlis, 2012).

The link between microsaccade behavior and arousal has previously been investigated in a number of different contexts. Increased microsaccade rates have been correlated with increased fatigue level, a clear mediator of arousal, as indexed by time-on-task (here referring to time spent performing the task) during virtual driving and tasks involving arithmetic calculation (Siegenthaler et al., 2014; Di Stasi et al., 2015). Moreover, the slope of the microsaccade main sequence (velocity/magnitude) has been found to decrease as time-on-task increases (Di Stasi et al., 2013, 2015). In other work, microsaccade rates have been found to increase before the onset of slow eye movements, which are another indicator of fatigue (Honda et al., 2014). Higher levels of light intensity may increase levels of alertness (i.e., decrease levels of fatigue), presumably through arousal-related mechanisms (Smolders et al., 2012). Consistent with this notion, a study found higher microsaccade rates under low luminance levels as compared to high luminance levels (Benedetto et al., 2014). Together, these findings suggest that microsaccade behavior is an effective index of fatigue-related arousal.

Arousal can also be influenced by the presentation of emotional stimuli. The relationship between emotional processing and microsaccade behavior has previously been investigated using emotional visual stimuli (Kashihara et al., 2014; Yep et al., 2018; Krejtz et al., 2020). Microsaccades occurring 300–600 ms after the brief presentation of emotional visual stimuli were significantly suppressed following the presentation of unpleasant stimuli as compared to pleasant or neutral stimuli (Kashihara et al., 2014). However, microsaccade rates measured at a similar epoch (250–300 ms after the presentation of stimuli) were not found to differ across different facial expressions of emotion (Yep et al., 2018). In another study, microsaccade rates and magnitudes were similar across neutral, aversive, and erotic stimuli conditions, though interactions between emotion and cognitive task manipulations were noted (Krejtz et al., 2020). Although some emotion modulations on microsaccade behavior have been described, the influence of emotional arousal on microsaccade behavior has yet to be systematically investigated. It remains to be determined whether emotional arousal modulates microsaccade rates and metrics in a manner similar to fatigue-related arousal.

Microsaccades are also modulated by task difficulty, another mediator of arousal (Gellatly and Meyer, 1992). Research has shown lower microsaccade rates and larger microsaccade magnitudes during more difficult tasks (Siegenthaler et al., 2014; Gao et al., 2015; Krejtzid et al., 2018). This modulation by cognitive processes has been extended to the pro- and anti-saccade paradigm (Watanabe et al., 2013; Yep et al., 2018; Dalmaso et al., 2020), in which subjects are instructed to either automatically look at the peripheral stimulus (pro-saccade) or to suppress this automatic response and voluntarily look in the opposite direction from the stimulus (anti-saccade). Here, microsaccade rates are lower during anti-saccade preparation. However, the modulation of saccade preparation on microsaccade metrics has yet to be investigated.

Evidently, arousal is a complex physiological process that is mediated by sensory (i.e., luminance), cognitive (i.e., task preparation), and affective (i.e., emotional stimuli) inputs. Although the modulation of fatigue-related arousal on microsaccade rates and dynamics has been well characterized, the influence of other aspects of arousal on microsaccade behavior is less understood. The goal of the present study is to investigate the influence of emotional arousal, saccade preparation, and global luminance level on microsaccade rates and dynamics by incorporating emotional auditory stimuli into an interleaved pro- and anti-saccade paradigm. We hypothesize that highly arousing auditory stimuli (as well as higher global luminance levels and anti-saccade preparation) will produce lower microsaccade rates and a steeper microsaccade main sequence slope. Our results show modulations of microsaccade behavior by saccade preparation and global luminance level, but not emotional auditory stimuli. These findings suggest differential modulations of microsaccade behavior by arousal mediated by different sensory, cognitive, and affective inputs.

## Materials and Methods

### Experimental Setup

All experimental procedures were reviewed and approved by the Institutional Review Board of the Taipei Medical University, Taiwan, and were in accordance with the Declaration of Helsinki (World Medical Association, 2001). Twenty-seven participants (mean age: 25.7, SD: 4 years, 14 males) were recruited via an advertisement posted on the Taipei Medical University website. Participants had normal or corrected-to-normal vision and were naïve regarding the purpose of the experiment. Participants provided informed consent and were compensated financially for their participation (∼US $10).

### Recording and Apparatus

Participants were seated in a dark room with their heads resting comfortably in a head rest. Eye position and pupil size were measured with a video-based eye tracker (Eyelink-1000 plus binocular-arm, SR Research, ON, Canada) at a rate of 500 Hz with binocular recording (left pupil was used). Stimulus presentation and data acquisition were controlled by Eyelink Experiment Builder and Eyelink software, as described previously (Hsu et al., 2020). Visual stimuli were presented on an LCD monitor at a screen resolution of 1920 × 1080 pixels (60 Hz refresh rate), subtending a viewing angle of 58°× 32°, with the distance from the eyes to the monitor set at 60 cm.

### Pro- and Anti-saccade Task With Emotional Auditory Stimuli (Experiment 1: Figure 1A)

A subset of the results, as well as a full description of the methods associated with Experiment 1 have been published previously with the focus being on pupil size and saccades (Cherng et al., 2020). Briefly, each trial began with the appearance of a central fixation point (FP; 0.5° diameter, gray color, 20 cd/m^2^) on a background (Dark: 2 cd/m^2^ or Bright: 16 cd/m^2^) in a dark room. After 500 ms of central fixation, an emotional auditory stimulus from the International Affective Digital Sounds (Bradley and Lang, 2007) was presented with the central FP for 6,000 ms. Afterward, though the overall luminance level did not change, the color of the FP changed in accordance with the saccade task condition (the luminance level of the two FP colors were matched, 20 cd/m^2^). On pro-saccade trials, participants were instructed to look towards the peripheral target stimulus as soon as it appeared. On anti-saccade trials, participants were instructed to look in the opposite direction of the target stimulus as soon as it appeared. After another 1,200 ms of central fixation, the FP disappeared for 200 ms (gap) before the peripheral target stimulus appeared (0.5° diameter, 270 cd/m^2^) to the left or right of the FP (8° eccentricity on the horizontal axis). This was followed by the presentation of two questions on the screen. Participants were asked to rate the degree of arousal and valence of the emotional auditory stimuli on nine-point scales using a keypad (Libby et al., 1973; Wang et al., 2018a). When rating arousal, 1 indicated a low degree of arousal and 9 indicated a high degree of arousal. When rating valence, 1 indicated an unpleasant stimulus, 5 indicated a neutral stimulus, and 9 indicated a pleasant stimulus. Participants came in twice (1 week apart) to complete the same experiment with two different background luminance levels (the order of the two background luminance level experiments was counterbalanced across participants). To ensure that each task condition included an equal number of each level of emotional valence, trials were separated into three valence categories (each category had 52 trials) according to previously-established rating values (Bradley and Lang, 2007). Each set of trials for a particular emotional valence contained 13 trials for each task condition (pro- and anti-saccade) and stimulus location (left and right). The experiment therefore consisted of 156 trials, in addition to 5 practice trials (which included two practice auditory stimuli not from the International Affective Digitized Sound), lasting approximately 80 min in total. Task condition and stimulus location were randomly interleaved. Saccades made toward the right and left direction were combined for data analysis.

**FIGURE 1:**
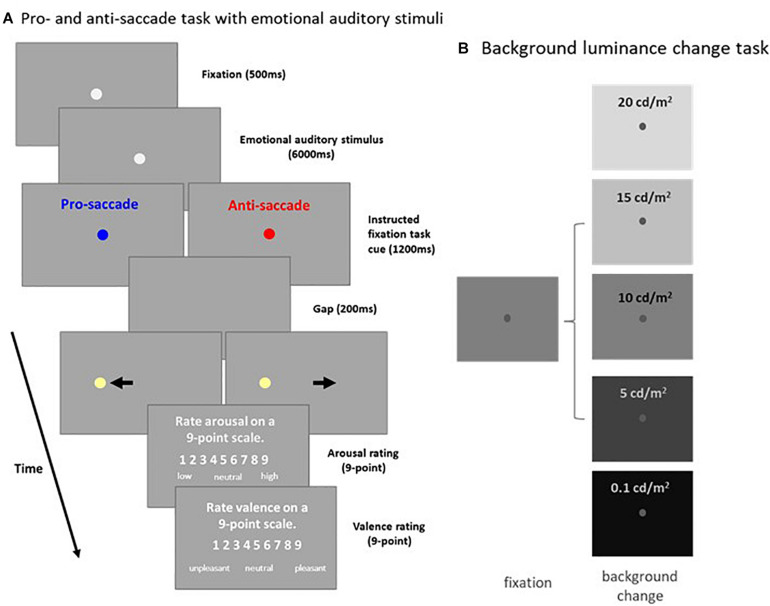
Experimental paradigms. **(A)** Each trial in the pro- and anti-saccade task with emotional auditory stimuli began with the appearance of a central FP on a background (Dark: 2 cd/m^2^ or Bright: 16 cd/m^2^). After a 500 ms delay, an emotional auditory stimulus was presented for 6 s, followed by the presentation of the instructed colored fixation cue (1,200 ms) for either the pro- or anti-saccade condition. A blank screen was presented for 200 ms (gap) before target stimulus presentation, and participants were then required to move their eyes toward the target (pro-saccade condition), or look away to the opposite location (anti-saccade condition). Following target stimulus offset, participants were required to answer two questions about the auditory stimulus presented. **(B)** Each trial in the background luminance change task began with the appearance of a central FP on a gray background (10 cd/m^2^). After 900–1,100 ms of fixation, the background luminance either increased (20 or 15 cd/m^2^), decreased (5 or 0.1 cd/m^2^), or stayed the same (10 cd/m^2^). Participants were required to maintain steady fixation for an additional 2,000–2,500 ms. FP: fixation point. Bkgd: background.

### Background Luminance Change Task (Experiment 2: Figure 1B)

Each trial began with the appearance of a central FP (0.5° diameter, 25 cd/m2) on a gray background (10 cd/m2). After 900–1,100 ms of central fixation, background luminance either increased to 15 or 20 cd/m^2^, decreased to 0.1 or 5 cd/m^2^ (both with 50 and 100% contrast relative to the gray background), or stayed the same (10 cd/m^2^). Participants were required to maintain steady fixation for an additional 2 – 2.5 s. Background luminance conditions were randomly interleaved, and each condition had 20 trials, lasting approximately 20 mins in total.

### Emotional Auditory Stimuli

Previous research has investigated the relationship between emotional processing and microsaccade behavior using visual stimuli (Kashihara et al., 2014; Yep et al., 2018; Krejtz et al., 2020). However, foveated visual information modulates fixation-related activity in the SC, which in turn affects microsaccade generation (Hafed, 2011; Hafed and Krauzlis, 2012). To avoid this potential confound associated with the use of visual stimuli, here we used auditory stimuli to induce emotional arousal. Audio clips were selected from the International Affective Digitized Sound (IADS) database system (Bradley and Lang, 2007). 8 clips were excluded due to short duration (< 6,000 ms; clips 104, 204, 275, 296, 365, 400, 698, and 699). Auditory stimuli were presented through headphones. The volume of the stimuli was fixed at a level predetermined to be comfortable and clear for all participants.

### Data Analysis

Aspects of analyses related to saccade and pupil behavior in Experiment 1 have been described in detail previously (Cherng et al., 2020). Briefly, in the saccade analysis for Experiment 1, trials were scored as correct if the first saccade after visual target appearance was in the correct direction (i.e., toward the target in the pro-saccade condition; away from the target in the anti-saccade condition). Direction errors were identified as the first saccade after target appearance in the wrong direction (i.e., toward the target in the anti-saccade condition). Consistent with the literature (Everling and Fischer, 1998; Munoz and Everling, 2004; Antoniades et al., 2013; Coe and Munoz, 2017; Hsu et al., 2020), higher direction errors and longer saccade reaction times were observed in the anti-saccade condition as compared to the pro-saccade condition. These results suggest that participants understood the task instructions, and that saccade preparation significantly modulated subsequent behavior. Trials with eye position deviation of more than 2° from the central FP or with detected saccades (>2° amplitude) during the required period of central fixation were excluded from analysis. Data from Experiment 1 during the dark background condition was removed for two participants due to the amount of blinking and presence of eye movements during the fixation period. These two participants were thus not recruited for the background luminance change experiment (Experiment 2). When blinks were detected, following the literature, pre- and post-blink pupil values were used to perform a linear interpolation to replace the pupil values lost during the blink period (Karatekin et al., 2010; Nassar et al., 2012; Wang et al., 2018b). Trials were discarded when two blinks occurred within a time interval of less than 500 ms. Participants with at least 5 microsaccade trials in each condition were included for data analysis, therefore, each analysis includes data from a different number of participants. Because of the smaller number of microsaccades that occurred during the background luminance change experiment, participants with two or more microsaccades in each condition were included.

Microsaccades were detected using a previously developed algorithm (Engbert and Kliegl, 2003; Engbert and Mergenthaler, 2006) that has been implemented in a number of recent studies (Watanabe et al., 2013; Yep et al., 2018; Wang et al., 2020). Briefly, the velocity threshold of fixational saccades was defined flexibly depending on the noise level of each trial (threshold: 6 SDs). The minimum duration for fixational saccades that exceeded the velocity threshold was set to 6 ms. This analysis was limited to a temporal period in which eye positions were relatively stable (i.e., the required period of central fixation). We only included microsaccades that occurred simultaneously in both eyes during at least one data sample (2 ms) in order to reduce the noise in data analyses. The magnitude (amplitude in degree), peak velocity (deg/s), and main sequence slope (peak velocity/magnitude) of microsaccades were analyzed. Microsaccade rate was first calculated on an individual participant basis (averaged across all trials in each condition), and then rates for the corresponding conditions were averaged across all participants. Following previous research (e.g., Engbert and Kliegl, 2003; Laubrock et al., 2005; Valsecchi and Turatto, 2009), the histogram of microsaccades was scaled to a rate-per-second measure, which was computed within a moving window of 100 ms.

Different epochs were used to capture microsaccade behavior for different analyses. In the time-on-task analysis, the whole period of central fixation with a visual fixation point was used (0 – 7,200 ms post-auditory-stimulus onset: 6,000 ms auditory stimulus presentation + 1,200 ms instructed fixation cue). In the emotional arousal (or valence) analysis, the epoch of 0 – 6,000 ms post-auditory-stimulus onset was used. In the saccade preparation analysis, the epoch of 0 – 1,400 ms post-instructed-cue onset was used. In the global luminance analysis, the epoch of 100 – 2,000 ms post-background-luminance change was used.

A one-way repeated-measure ANOVA was used to examine the effects of experimental conditions on microsaccade behavior (rates and dynamics: slope, velocity, and magnitude) and Bonferroni-corrected *t*-tests were used for the planned comparisons, except where indicated. A two-tailed student *t*-test was performed to compare the differences between the two conditions. Effect sizes, where appropriate, are also reported. Statistical tests were performed using JASP Team (2019). Furthermore, the impact of emotional arousal, saccade preparation, and background luminance on microsaccade dynamics was further analyzed with linear mixed models that allowed us to include these variables as fixed effects in addition to a random term to capture idiosyncratic differences across participants (Pinheiro and Bates, 2000).

## Results

### Emotional Arousal Effects on Microsaccade Behavior

**FIGURE 2:**
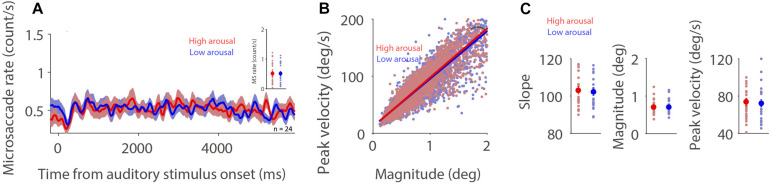
Effect of emotional arousal on microsaccade behavior. Microsaccade behavior following presentation of auditory stimuli in two emotional arousal conditions (High and Low) under the dark background condition **(A–C)**. Mean microsaccade rates (0 to 6,000 ms) **(A)**, microsaccade main sequence **(B),** and microsaccade main sequence slope, magnitude, and peak velocity **(C)** shown for different emotional arousal conditions under the dark background condition. **(A)** The shaded colored regions surrounding the microsaccade rate curves represent the ± standard error range (across participants) for different conditions. **(A,C)** The large-circle and error-bars represent the mean values ± standard error across participants. The small circles represent the mean value for each participant. **(B)** Solid lines indicate the linear regression lines. *Indicates differences are statistically significant.

If, as argued, the influence of fatigue on microsaccade behavior is associated with arousal level (Siegenthaler et al., 2014; Di Stasi et al., 2015), highly arousing sounds should produce a lower microsaccade rate and a steeper microsaccade main sequence slope. To understand whether microsaccade dynamics are modulated by emotional arousal evoked by auditory stimuli, we separated trials into two arousal categories (high: arousal > the median arousal value of a given participant; low: arousal < the median arousal value of a given participant) in order to have an approximately equal number of trials across conditions and participants. As displayed in Figure 2A, microsaccade rates under the dark background condition were similar between the two arousal levels (0–6,000 ms post-auditory-stimulus onset; *t*(23) = 0.0994; *p* = 0.92; *d* = 0.026). The relationship between microsaccade peak velocity and magnitude in the two arousal conditions with trials collapsed across participants was observed (Figure 2B, high and low arousal: *R* = 0.89, 0.89; all *p*s < 0.001). Microsaccade dynamics were not modulated by emotional arousal level (Figure 2C, slope: *t*(23) = 0.907, *p* = 0.374, *d* = 0.185; magnitude: *t*(23) = 0.453, *p* = 0.655, *d* = 0.093; peak velocity: *t*(23) = 0.984, *p* = 0.335, *d* = 0.201). The same pattern of results was observed under the bright background condition (Supplementary Figure 1).

A linear mixed model was used to investigate the influence of emotional arousal and background luminance on microsaccade dynamics, taking emotional valence into account. This model included emotional arousal (1–9), emotional valence (1–9), and background luminance (Dark: 1; Bright: 2) as fixed predictors. The linear mixed model was as follows:

$$M\,o\,d\,e\,l\,1:y=\beta_{0}+\beta_{S}+\beta_{1}\,A+\beta_{2}\,V+\beta_{3}\,L$$

where *A* is emotional arousal, *V* is emotional valence, *L* is background luminance level, β_*S*_ is a Gaussian random variable fitted for each participant as an individual offset, and β_*i*_ are the standard coefficients of the statistical model (intercept and slopes). For microsaccade main sequence slope, β_1_ = 0.0245, *p* = 0.6842; β_2_ = 0.0701, *p* = 0.2267; β_3_ = −0.3227, *p* = 0.1509 (see details in Supplementary Table 1). For microsaccade magnitude, β_1_ = 0.0013, *p* = 0.2077; β_2_ = −8.76E-04, *p* = 0.3838; β_3_ = 0.0386, *p* = 0.0463. For microsaccade peak velocity, β_1_ = 0.1206, *p* = 0.2098; β_2_ = 0.0182, *p* = 0.8446; β_3_ = 3.3471, *p* = 1.15E-20. Together, these results suggest that in the current paradigm, when emotional valence is taken into account, emotionally arousing auditory stimuli do not modulate microsaccade behavior. The effect of emotional valence on microsaccade behavior is appended in Supplementary Figure 2.

Note that the effect of fatigue on microsaccade behavior is appended in Supplementary Figure 3. Consistent with the literature (Siegenthaler et al., 2014; Di Stasi et al., 2015), our results showed increased microsaccade rates and decreased microsaccade main sequence slope as time-on-task increased. This pattern of results was particularly pronounced under the dark background condition.

### Saccade Preparation Effects on Microsaccade Behavior

**FIGURE 3:**
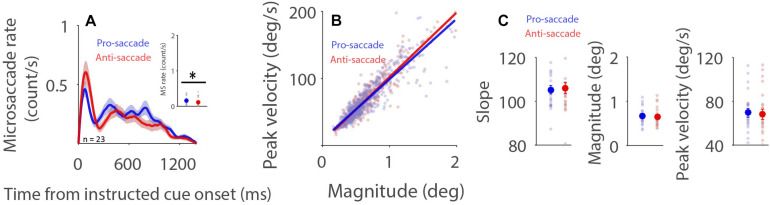
Effect of saccade preparation on microsaccade behavior. Microsaccade behavior following the onset of the instructive fixation cue for different saccade preparation conditions (pro and anti-saccade) under the dark background condition **(A–C)**. Mean microsaccade rates (600 to 1,400 ms) **(A)**, microsaccade main sequence **(B),** and microsaccade main sequence slope, magnitude, and peak velocity **(C)** shown for different saccade preparation conditions under the dark background condition. **(A)** The shaded colored regions surrounding the microsaccade rate curves represent the ± standard error range (across participants) for different conditions. **(A,C)** The large-circle and error-bars represent the mean values ± standard error across participants. The small circles represent the mean value for each participant. **(B)** Solid lines indicate the linear regression lines. ^∗^Indicates differences are statistically significant.

Microsaccade behavior has been shown to be modulated by task difficulty (Siegenthaler et al., 2014; Gao et al., 2015; Xue et al., 2017; Krejtzid et al., 2018), with decreased microsaccade rates and generally increased microsaccade magnitudes being observed during more difficult task conditions. It is argued (Siegenthaler et al., 2014) that attention to task-related processes is increased during more difficult conditions, resulting in less attention being directed toward the maintenance of fixation. Decreased attention toward fixation causes lower and broader rostral SC activity, which in turn produces lower microsaccade rates and larger microsaccade magnitudes. If, as proposed, microsaccade rates increase and microsaccade magnitudes decrease with higher rostral activity, then reduced microsaccade rates and increased microsaccade magnitudes should be observed during preparation for pro-saccades as compared to anti-saccades, because rostral SC activity is increased during anti-saccade preparation (Everling et al., 1999). As displayed in Figure 3A, microsaccade rates under the dark background condition generally decreased after the onset of the instructed fixation cue for saccade preparation. Moreover, microsaccade rates were lower during preparation for anti-saccades than that for pro-saccades (Figure 3A, 600–1,400 ms post-fixation-cue onset: pro: 0.161 ± 0.022; anti: 0.119 ± 0.024; *t*(22) = 2.225; *p* = 0.037; *d* = 0.464), consistent with previous studies using the pro- and anti-saccade paradigm (Watanabe et al., 2013; Yep et al., 2018; Dalmaso et al., 2020). A robust relationship between peak velocity and magnitude was observed during pro- and anti-saccade preparation (Figure 3B, pro: *R* = 0.9; anti: *R* = 0.92, all *p*s < 0.001). Microsaccade dynamics were not modulated by saccade preparation under the dark background condition (Figure 3C, slope: *t*(22) = 0.415, *p* = 0.682, *d* = 0.086; magnitude: *t*(22) = 1.235, *p* = 0.230, *d* = 0.258; peak velocity: *t*(22) = 0.760, *p* = 0.456, *d* = 0.158). The same pattern of results was observed under the bright background condition (Supplementary Figure 4).

Similarly, a linear mixed model was used to further investigate the influence of saccade preparation and background luminance on microsaccade behavior. This model included saccade preparation (Pro: 1; Anti: 2), background luminance (Dark: 1; Bright: 2), and saccade preparation × background luminance as fixed predictors. The linear mixed model was as follows:

$$M\,o\,d\,e\,l\,2:y=\beta_{0}+\beta_{S}+\beta_{1}\,S+\beta_{2}\,L+\beta_{3}\,S*L$$

where *S* is saccade preparation, *L* is background luminance level, β_*S*_ is a Gaussian random variable fitted for each participant as an individual offset, and β_*i*_ are the standard coefficients of the statistical model (intercept and slopes). For microsaccade slope, β_1_ = −0.302, *p* = 0.899; β_2_ = −1.756, *p* = 417; β_3_ = 0.131, *p* = 0.926. No modulation was observed in magnitude or peak velocity results (see details in Supplementary Table 2). Together, these results suggest that saccade preparation modulates microsaccade rate, but not microsaccade main sequence slope, magnitude or velocity. The lower microsaccade rates and larger microsaccade magnitudes previously observed during more difficult task conditions may not be mediated by rostral SC activity, as suggested previously (Siegenthaler et al., 2014).

### Global Luminance Effects on Microsaccade Behavior

**FIGURE 4:**
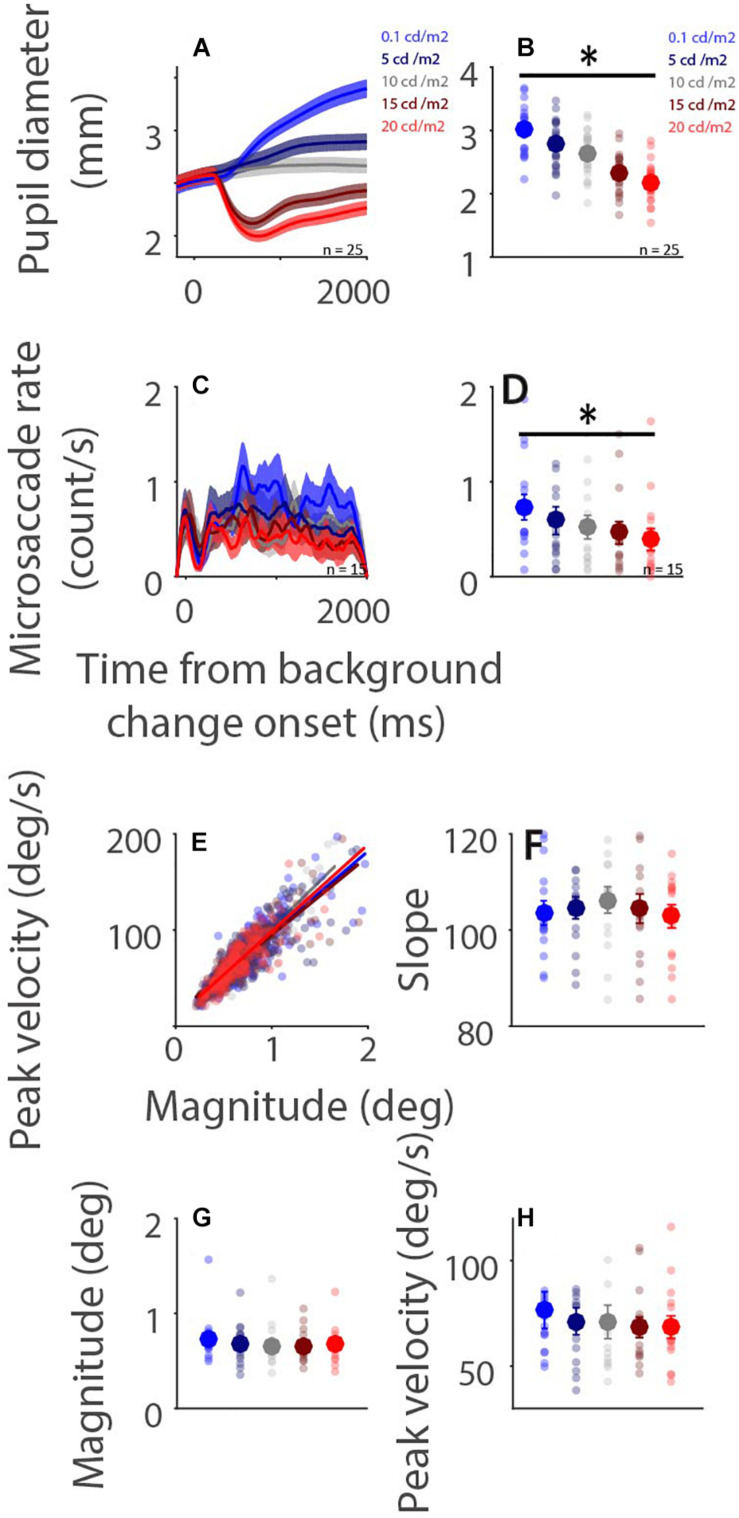
Effect of background luminance level on microsaccade behavior. Pupil diameter and microsaccade rates following background luminance change under different conditions **(A,C)**. Mean pupil diameter (100–2,000 ms) **(B)**, mean microsaccade rates (100–2,000 ms) **(D)**, microsaccade main sequence **(E)**, main sequence slope **(F)**, magnitude **(G)**, and peak velocity **(H)** shown for different background luminance conditions. **(A,C)** The shaded colored regions surrounding the response curves represent the ± standard error range (across participants) for different conditions. **(B,D,F,G,H)** The large-circle and error-bars represent the mean values ± standard error across participants. The small circles represent the mean value for each participant. epochs. **(E)** Solid lines indicate the linear regression lines. ^∗^ indicates differences are statistically significant.

Higher levels of light intensity increase levels of alertness (Smolders et al., 2012), and could therefore also influence microsaccade behavior via arousal-related mechanisms. Although the results of Experiment 1 demonstrate some modulations of background luminance on microsaccade behavior, these results could be confounded by ongoing affective and/or cognitive processes. To more directly examine global luminance effects on microsaccade rate and metrics, participants were required only to maintain central fixation while background luminance was altered in Experiment 2 (Figure 1B). Pupil diameter dynamics are displayed in Figure 4A, showing that pupil size changes as a function of background luminance level (Figure 4B: pupil diameter 100–2,000 ms after background luminance change: low to high luminance level: 2.994 ± 0.080, 2.755 ± 0.084, 2.620 ± 0.078, 2.312 ± 0.070, 2.175 ± 0.066 mm; *F*(4,84) = 308.552, *p* < 0.001, η_*p*_^2^ = 0.936). Microsaccade rates were also modulated by global luminance level (Figure 4C), with lower rates observed during higher levels of luminance (Figure 4D: epoch of 100–2,000 ms: low to high luminance level: 0.731 ± 0.130, 0.591 ± 0.140, 0.523 ± 0.121, 0.463 ± 0.114, 0.391 ± 0.113; *F*(4,56) = 10.689, *p* < 0.001, η_*p*_^2^ = 0.433). These results were consistent with previous research, showing lower microsaccade rates under high screen luminance (Benedetto et al., 2014). In addition, because there were no differences between the change (pupillary light reflex: highest and 2nd highest luminance level conditions; pupillary darkness reflex: lowest and 2nd lowest luminance level conditions) and no change conditions (no changes in background luminance), these results suggest that the pupillary light and darkness reflex do not affect microsaccade rates. A robust relationship between microsaccade peak velocity and magnitude was observed under all luminance conditions with trials collapsed across participants (Figure 4E, low to high luminance level R values: 0.86, 0.87, 0.91, 0.86, and 0.87, all *p*s < 0.001). The microsaccade main sequence slope was not modulated by global luminance level [Figure 4F: *F*(4,56) = 0.619, *p* = 0.651, η_*p*_^2^ = 0.042]. Though microsaccade magnitude and peak velocity decreased with increasing luminance level, these results were not statistically significant [Figure 4G: magnitude: *F*(4,56) = 1.730, *p* = 0.156, η_*p*_^2^ = 0.110; Figure 4H: peak velocity: *F*(4,56) = 1.436, *p* = 0.255, η_*p*_^2^ = 0.093].

To further quantify the results, a linear mixed model was used. The predictor was background luminance level (Dark−*Bright*: 1−5). The linear mixed model was as follows:

$$M\,o\,d\,e\,l\,3:y=\beta_{0}+\beta_{S}+\beta_{1}\,L$$

where *L* is background luminance level, β_*S*_ is a Gaussian random variable fitted for each participant as an individual offset, and β_*i*_ are the standard coefficients of the statistical model (intercept and slopes). For microsaccade slope, β_1_ = 0.324, *t*(1617) = 1.06, *p* = 0.289. For microsaccade magnitude, β_1_ = −0.013, *t*(1617) = −2.936, *p* = 0.0034. For microsaccade peak velocity, β_1_ = −1.289, *t*(1617) = −3.088, *p* = 0.0021. Together, these results suggest that global luminance level modulated microsaccade rates, but not microsaccade main sequence slope.

## Discussion

The current study systematically examined the effects of emotional arousal, saccade preparation, and global luminance level on microsaccade behavior. Microsaccade behavior was found to be significantly modulated by saccade preparation and global luminance level, but not emotional arousal. In the pro- and anti-saccade task (Experiment 1), microsaccade rate was lower during anti-saccade preparation as compared to pro-saccade preparation, though microsaccade dynamics were comparable during both trial types. The incorporation of emotional auditory stimuli prior to instructed saccade cue onset in this paradigm did not result in any changes in microsaccade rate or dynamics across different emotional arousal levels. In the background luminance change task (Experiment 2), increased microsaccade rates were observed under lower levels of global luminance. In summary, these results reveal differential modulation of microsaccade behavior by arousal linked to emotion, fatigue, saccade preparation, and global luminance level. Moreover, our results suggest that the modulation of task difficulty on microsaccade behavior cannot be explained by increased rostral SC activity during more difficult conditions, as previously suggested.

### Emotional Arousal Effects on Microsaccade Behavior

The influence of emotional processing on microsaccade behavior has previously been examined, and has shown decreased microsaccade rates after the brief presentation of unpleasant emotional stimuli as compared to pleasant or neutral emotional stimuli (Kashihara et al., 2014). However, in other studies, no differences in microsaccade rates or dynamics were observed across different emotion conditions (Yep et al., 2018; Krejtz et al., 2020). Inconsistent results could be due to differences between explicit and implicit emotional processing (Critchley et al., 2000; Winston et al., 2003; D’Hondt et al., 2016), with weaker emotional modulation being evoked when executive control is involved (Cohen et al., 2016). Due to the brief presentation of visual stimuli in the work of Kashihara et al. (2014), emotional responses might have been more pronounced. In contrast to previous studies which have investigated the influence of different emotions on microsaccade behavior using visual stimuli, here we focused on emotional arousal modulation using auditory stimuli. Emotional arousal induced by emotional auditory stimuli did not modulate microsaccade rate, main sequence slope, magnitude, or velocity. Moreover, the regression model, which allowed us to investigate the influence of emotional arousal while also taking emotional valence into account, revealed no clear modulation of emotional arousal on microsaccade behavior. Although it is possible that our statistical power was too weak to reveal emotional arousal effects on microsaccade behavior, as indicated by small values 5–8% of the power analysis (one-sided test, with alpha value 0.05), Bayesian *t* test (scale factor *r* = 0.707) (Rouder et al., 2009) on emotional arousal effects showed that BF_10_ values ranged between 0.021 and 0.033, providing substantial evidence in favor of the null model (Wetzels et al., 2011).

### Fatigue-Related Arousal Effects on Microsaccade Behavior

Fatigue-related arousal has consistently been shown to influence microsaccade behavior. Previous research has found that microsaccade rates increase with increasing fatigue level, as indexed by time-on-task (Siegenthaler et al., 2014; Di Stasi et al., 2015), although one study has reported a decrease in microsaccade rate with increasing time-on-task (Di Stasi et al., 2013). Moreover, it has been shown that microsaccade main sequence slope decreases with time-on-task (Di Stasi et al., 2013, 2015). The modulation of microsaccade behavior by fatigue-related arousal is also supported by studies demonstrating increased microsaccade rates before the onset of slow eye movements and under low levels of screen luminance, two additional indices of fatigue, and alertness (Benedetto et al., 2014; Honda et al., 2014). Consistently, here we found that microsaccade rates increased, and microsaccade main sequence slope decreased with increasing time-on-task, and further, that microsaccade rates were lower under higher luminance levels. These findings therefore support and contribute to previous work demonstrating the influence of fatigue-related arousal on microsaccade behavior.

### Task Difficulty and Saccade Preparation Effects on Microsaccade Behavior

Task difficulty has also been shown to affect microsaccade rates and dynamics. Decreased microsaccade rates and increased magnitudes are often observed during more demanding task conditions (Siegenthaler et al., 2014; Gao et al., 2015; Xue et al., 2017; Krejtzid et al., 2018), although increased microsaccade rates in dual task conditions (Benedetto, 2011), and smaller microsaccade magnitudes under high perceptual load conditions (Xue et al., 2017) have also been reported. Though rostral SC activity plays a central role in generating microsaccades (Hafed, 2011), its proposed role in task difficulty modulation on microsaccade behavior (Siegenthaler et al., 2014) is not supported by the results from the interleaved pro- and anti-saccade paradigm described here. Consistent with previous studies (Watanabe et al., 2013; Yep et al., 2018; Dalmaso et al., 2020), we found lower microsaccade rates during anti-saccade preparation compared to pro-saccade preparation. Given the higher rostral SC activity observed during anti-saccade preparation (Everling et al., 1999; Munoz and Everling, 2004), our results suggest that higher rostral SC activity suppresses microsaccade generation. Microsaccade magnitudes were similar between preparation of pro- and anti-saccades, further suggesting that microsaccade magnitudes may not be significantly modulated by the level of rostral SC activity. Alternatively, because task difficulty is also linked to arousal (Gellatly and Meyer, 1992), it is possible that more difficult tasks evoke higher arousal levels that affect microsaccades, reducing microsaccade rates in the anti-saccade condition. However, this account still cannot explain similar microsaccade main sequence slopes between pro- and anti-saccade preparation, if higher arousal should produce a steeper slope. Together, it remains to be determined which mechanisms mediate the influence of task difficulty on microsaccade behavior.

### Limitations and Future Directions

Cognitive and emotional processes are both influenced by age (Mroczek, 2001; Salthouse, 2010; Urry and Gross, 2010). The frontal-temporal network has previously been implicated in age-related compensatory mechanisms for executive functioning during oculomotor paradigms (e.g., Nelles et al., 2009; Fernandez-Ruiz et al., 2018). Therefore, changes in executive functioning associated with aging should affect the modulation of microsaccade behavior in the pro- and anti-saccade paradigm. Since emotion regulation is also modulated by age (Mroczek, 2001; Urry and Gross, 2010), our understanding of microsaccade behavior would benefit from future studies which investigate the influences of age, cognitive control, and emotion regulation on microsaccade rate and metrics. The current study is limited by the characterization of microsaccade behavior in a relatively small study cohort consisting only of young adults. Future work using larger study cohorts which span a greater age range are required to adequately characterize the influence and interaction of the processes described above.

## Conclusion

Arousal is a complex physiological process that is modulated by various sensory, cognitive, and affective inputs. As more studies are using microsaccade behavior to index higher cognitive processes and dysfunction in neurological and neuropsychiatric disease, characterizing the influence of arousal on microsaccade rate and dynamics has become increasingly important. The current study demonstrates a clear modulation of microsaccade behavior by task preparation, global luminance level, and time-on-task, but not emotional arousal. Given that these processes likely interact in a number of complex and context-dependent ways, future work aimed at systematically characterizing each aspect of arousal, as well as their joint influence on task performance, will be critical to fully understand arousal effects in healthy and clinical human populations.

## Data Availability Statement

The raw data supporting the conclusions of this article will be made available by the authors, without undue reservation.

## Ethics Statement

The studies involving human participants were reviewed and approved by Institutional Review Board of the Taipei Medical University, Taiwan. The patients/participants provided their written informed consent to participate in this study.

## Author Contributions

J-TC, Y-GC, and Y-FH performed research. C-AW designed research, analyzed data, and wrote the manuscript. All authors contributed to the study conception and provided comments and edits on various drafts of the manuscript.

## Conflict of Interest

The authors declare that the research was conducted in the absence of any commercial or financial relationships that could be construed as a potential conflict of interest.
