# Supplementary material for: Investigating Arousal, Saccade Preparation, and Global Luminance Effects on Microsaccade Behavior
Source: Front Hum Neurosci. 2021 Mar 5;15:602835. doi: 10.3389/fnhum.2021.602835 (PMC7973374; doi:10.3389/fnhum.2021.602835)
Supplement: Supplementary file 1 [file Data_Sheet_1.PDF]

Title:

Investigating arousal, anti-saccade preparation, and global luminance effects on microsaccade behavior

Jui-Tai Chen, Rachel Yep, Yu-Fan Hsu, Yih-Giun Cherng and Chin-An Wang

Supplementary materials

Supplementary Figure 1

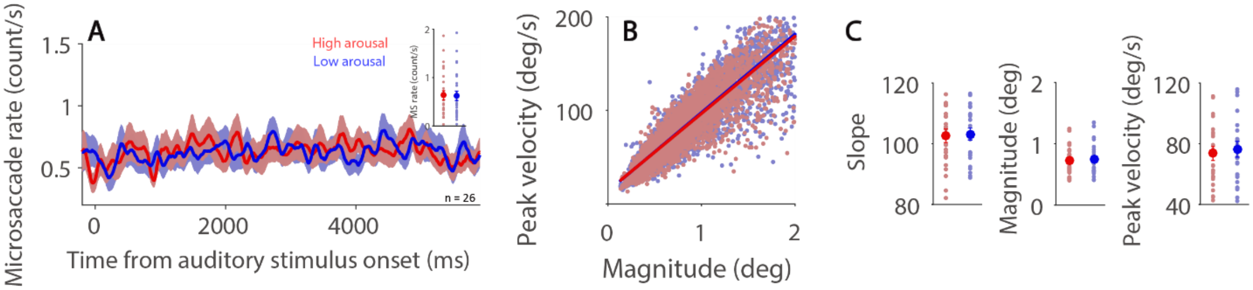

Effect of emotional arousal on microsaccade behavior. Microsaccade behavior following presentation of auditory stimuli in two emotional arousal conditions (High and Low) under the bright background condition **(A-C)**. Mean microsaccade rates (0 to 6000 ms) **(A)**, microsaccade main sequence **(B),** and microsaccade main sequence slope, magnitude, and peak velocity **(C)** shown for different emotional arousal conditions under the bright background condition. Similar microsaccade rates (A $t(25) = 0.864$, $p = 0.396$, $d = 0.169$) and dynamics (C slope: $t(25) = 0.622$, $p = 0.539$, $d = 0.122$; magnitude: $t(25) = 1.263$, $p = 0.218$, $d = 0.248$; peak velocity: $t(25) = 1.342$, $p = 0.192$, $d = 0.263$) were observed between the two arousal conditions, and a clear main sequence between microsaccade peak velocity and magnitude in the two arousal conditions (B high and low arousal: $R = 0.89, 0.90$; all $ps < 0.001$). In A, the shaded colored regions surrounding the microsaccade rate curves represent the ± standard error range (across participants) for different conditions. In A,C, the large-circle and error-bars represent the mean values ± standard error across participants. The small circles represent the mean value for each participant. In B, solid lines indicate the linear regression lines.

Supplementary Figure 2

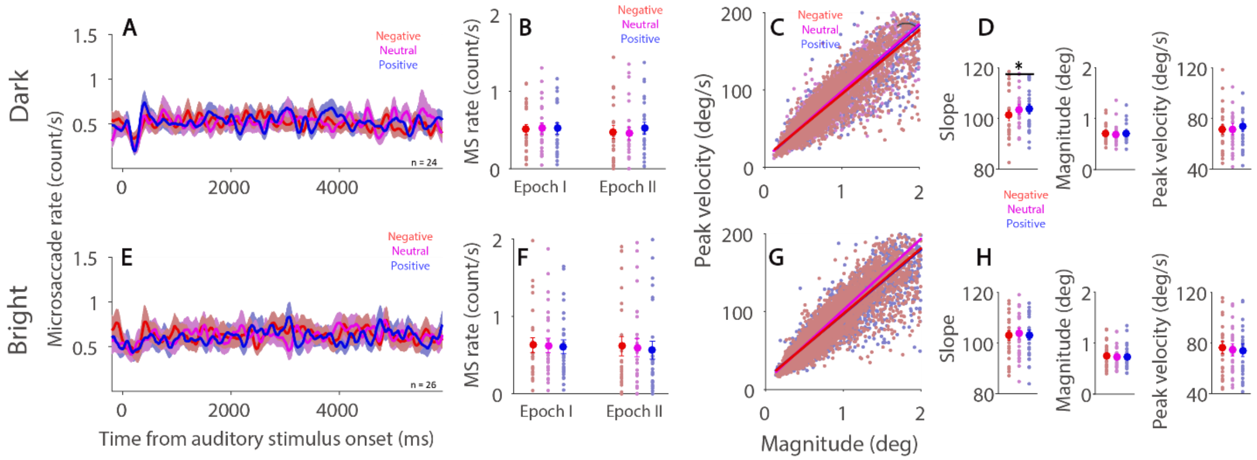

Effect of emotional valence on microsaccade behavior. To investigate the influence of emotional valence on microsaccade behavior induced by emotional auditory stimuli we separated trials into three valence categories (negative: valence < 5; neutral: valence = 5; positive: valence > 5) according to the subjective valence ratings on a trial-by-trial basis. As displayed in A, microsaccade rates under the dark background were similar across the three valence conditions (B: Epoch I: 1-6000 ms post-auditory-stimulus onset; $F(2,46) = 0.484$; $p = 0.620$, $\eta_p^2 = 0.021$). Microsaccade rates were also similar during the epoch of enhancement (B: Epoch II: 200 - 500 ms post-auditory-stimulus onset; $F(2,46) = 1.180$; $p = 0.317$, $\eta_p^2 = 0.049$). The relationship between microsaccade peak velocity and magnitude in the three valence conditions with trials collapsed across participants was observed (C, negative, neutral, positive valence: $R = 0.89$, 0.89, 0.88, all ps < 0.001). Microsaccade main sequence slope was modulated by emotional valence (D: $F(2,46) = 3.79$; $p = 0.046$, $\eta_p^2 = 0.141$), but microsaccade magnitude and peak velocity was not (D: magnitude: $F(2,46) = 1.509$; $p = 0.232$, $\eta_p^2 = 0.062$; peak velocity: $F(2,46) = 1.708$; $p = 0.193$, $\eta_p^2 = 0.069$). Similar patterns of results were observed under the bright background condition (E), with similar microsaccade rates (F, Epoch I: 1-6000 ms post-auditory-stimulus onset; $F(2,50) = 0.402$; $p = 0.671$, $\eta_p^2 = 0.016$; Epoch II: 200 - 500 ms post-auditory-stimulus onset; $F(2,50) = 0.579$; $p = 0.564$, $\eta_p^2 = 0.023$) and dynamics (H, slope: $F(2,50) = 0.755$; $p = 0.475$, $\eta_p^2 = 0.029$) being observed across the three valence conditions, though microsaccade magnitude and peak velocity were marginally modulated by emotional valence (magnitude: $F(2,50) = 3.259$; $p = 0.060$, $\eta_p^2 = 0.115$; peak velocity: $F(2,50) = 3.19$; $p = 0.069$, $\eta_p^2 = 0.113$). The main sequence relationship between microsaccade peak velocity and magnitude was observed across the three valence conditions (G). Because, as described in the manuscript, the

regression model showed no modulations on microsaccade dynamics by emotional valence, the effects observed here need to be interpreted with caution. In A and D the shaded colored regions surrounding the microsaccade rate curves represent the ± standard error range (across participants) for different conditions. In A,C,D,F, the large-circle and error-bars represent the mean values ± standard error across participants. The small circles represent the mean value for each participant. In B and E, solid lines indicate the linear regression lines. * indicates differences are statistically significant.

Supplementary Figure 3

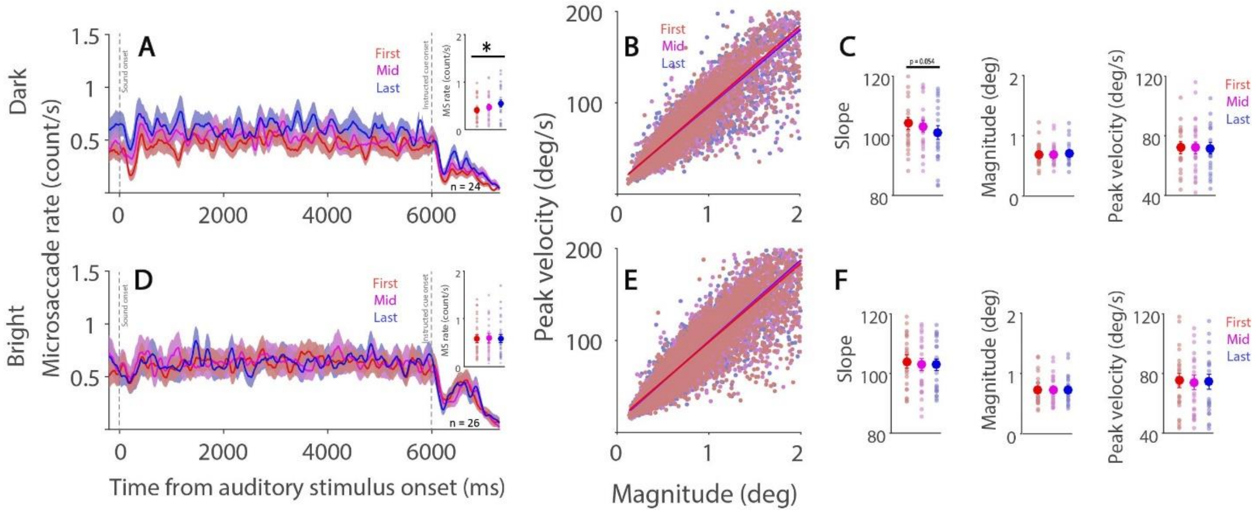

Effect of fatigue on microsaccade behavior. To investigate the influence of time-on-task on microsaccade behaviour, trials were separated into three temporal trial sequence categories with each category consisting of 52 trials (one third of the total trials) and lasting approximately 20-25 min (First trial group: trials 1-52; Mid trial group: trials 53-104; and Last trial group: trials 105-156). As illustrated in A, consistent with the literature (Rolfs, 2009; Valsecchi and Turatto, 2009; Hafed, 2011; Martinez-Conde et al., 2013), microsaccade rates decreased after the presentations of both auditory and visual stimuli (two dotted vertical lines) when presented under the dark background. Furthermore, microsaccade rates were modulated by time-on-task (i.e. trial sequence), with mean microsaccade rates during the epoch of 0-7200 ms post-auditory-stimulus onset being 0.407 ± 0.056 (mean ± s.e.m), 0.469 ± 0.060, and 0.549 ± 0.072 count/s in the First, Mid, and Last trial group category, respectively. In agreement with previous research (Siegenthaler et al., 2014; Di Stasi et al., 2015), microsaccade rates significantly increased with increasing time-on-task (A, $F(2,46) = 10.546$, $p = 0.002$, $\eta_p^2 = 0.314$; post-hoc with Bonferroni-corrected: First vs Mid, and First vs Last, ps < 0.05). As displayed inB, the microsaccade main sequence was revealed in three trial sequence categories with trials collapsed across participants (B, First, Mid, Last trial group category: R = 0.89, 0.88, 0.88; all $p < 0.001$). More importantly, microsaccade dynamics were also modulated by time-on-task, as documented previously (Di Stasi et al., 2013, 2015). The microsaccade main sequence slope decreased over time, although it was only marginally significant (C, $F(2,46) = 3.107$, $p = 0.054$, $\eta_p^2 = 0.119$; First vs Last, p = 0.057), with mean slope being 104.13 ± 9.83, 103.20 ± 9.25, and 101.09 ± 11.13 velocity/magnitude in the First, Mid, and Last trial group categories, respectively. Microsaccade

magnitude and peak velocity were not modulated by time-on-task (C, magnitude: $F(2,46) = 0.144$, $p = 0.866$, $\eta_p^2 = 0.006$; peak velocity: $F(2,46) = 0.010$, $p = 0.99$, $\eta_p^2 < 0.001$). Effects of time-on-task were not pronounced, however, with the bright background. Microsaccade rates were not systematically modulated by trial sequence (D, $F(2,50) = 0.059$, $p = 0.92$, $\eta_p^2 = 0.002$; mean microsaccade rates: 0.575 ± 0.082, 0.588 ± 0.100, and 0.581 ± 0.082 count/s in the First, Mid, and Last trial group category). Although the relationship between microsaccade peak velocity and magnitude was demonstrated in E (First, Mid, Last trial group categories: $R = 0.89$, 0.9, 0.9; all ps < 0.001), microsaccade dynamics were not modulated by time-on-task (F, slope: $F(2,50) = 0.592$, $p = 0.497$, $\eta_p^2 = 0.023$; magnitude: $F(2,50) = 0.144$, $p = 0.826$, $\eta_p^2 = 0.006$; peak velocity: $F(2,50) = 0.492$, $p = 0.576$, $\eta_p^2 = 0.019$).

The linear mixed model was as follows:

Model: $$y = \beta_0 + \beta_S + \beta_1 T + \beta_2 L + \beta_3 T * L$$

where T is trial sequence, L is background luminance level, β_S is a Gaussian random variable fitted for each participant as an individual offset, and β_i are the standard coefficients of the statistical model (intercept and slopes). For microsaccade slope, $\beta_1 = -0.04$, p = 1.08E-07; $\beta_2 = -1.75$, p = 1.07E-04; $\beta_3 = 0.014$, p = 0.0029 (see details in following Table). For microsaccade magnitude, $\beta_1 = 1.08E-04$, p = 0.4073; $\beta_2 = 0.0441$, p = 1.71E-08; $\beta_3 = -7.55E-05$, p = 0.339. For microsaccade peak velocity, $\beta_1 = -0.0181$, p = 0.132; $\beta_2 = 2.7954$, p = 1.06E-04; $\beta_3 = 0.0037$, p = 0.61. In summary, these results showed that the effect of time-on-task on microsaccade main sequence slope were different between the two background luminance levels, and as predicted, slope negatively correlated with trial number. These results together suggest that microsaccade behavior is indicative of arousal related to fatigue, particularly under a dark background condition.

Slope (vel/mag)	Regression coefficient	SE	*t*	*d.f.*	*p*
'(Intercept)'	109.81	1.86	59.09	31014	0.0000
'trial'	-0.04	0.01	-5.31	31014	0.0000
'bkgd'	-1.75	0.45	-3.87	31014	0.0001
'trial:bkgd'	0.01	0.00	2.98	31014	0.0029

Magnitude (deg)	Regression coefficient	SE	*t*	*d.f.*	*p*
'(Intercept)'	0.65	0.04	15.53	31014	0.0000

'trial'	0.00	0.00	0.83	31014	0.4073
'bkgd'	0.04	0.01	5.64	31014	0.0000
'trial:bkgd'	0.00	0.00	-0.96	31014	0.3390

Peak Velocity (deg/s)	Regression coefficient	SE	*t*	*d.f.*	*p*
'(Intercept)'	69.93	4.39	15.94	31014	0.0000
'trial'	-0.02	0.01	-1.51	31014	0.1320
'bkgd'	2.80	0.72	3.88	31014	0.0001
'trial:bkgd'	0.00	0.01	0.51	31014	0.6100

Supplementary Figure 4

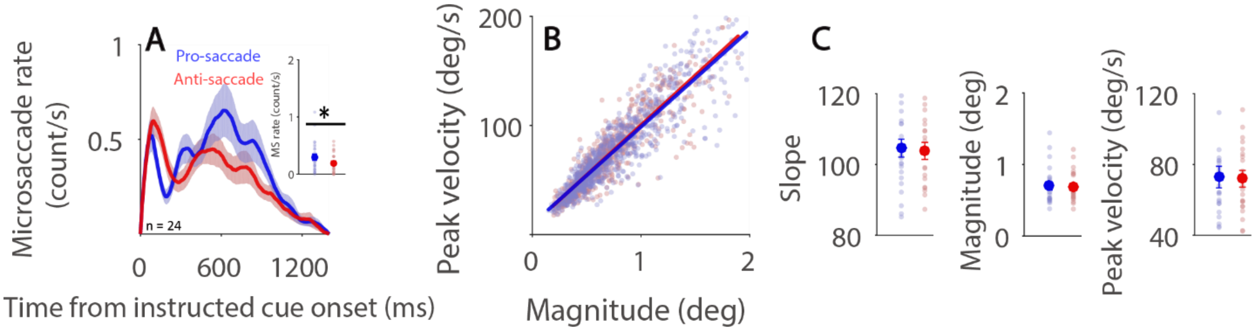

Effect of saccade preparation on microsaccade behavior. Microsaccade behavior following the onset of the instructive fixation cue for different saccade preparation conditions (pro and anti-saccade) under the bright background condition **(A-C)**. Mean microsaccade rates (600 to 1400 ms) **(A)**, microsaccade main sequence **(B)**, and microsaccade main sequence slope, magnitude, and peak velocity **(C)** shown for different saccade preparation conditions under the bright background. Lower microsaccade rates in the anti-saccade condition compared to the pro-saccade condition (A, 600-1400 ms post-fixation-cue onset: pro: 0.296 ± 0.057; anti: 0.195 ± 0.035; $t(22) = 3.064$; $p = 0.005$; $d = 0.625$). A clear relationship between microsaccade peak velocity and magnitude was also observed during pro- and anti-saccade preparation (B, pro: $R = 0.91$; anti: $R = 0.9$, all $ps < 0.001$). Microsaccade dynamics were not modulated by saccade preparation under the bright background condition (C, slope: $t(25) = 0.505$, $p = 0.618$, $d = 0.103$; magnitude: $t(25) = 0.260$, $p = 0.797$, $d = 0.053$; peak velocity: $t(25) = 0.385$, $p = 0.703$, $d = 0.079$). In A, the shaded colored regions surrounding the microsaccade rate curves represent the ± standard error range (across participants) for different conditions. In A,C, the large-circle and error-bars represent the mean values ± standard error across participants. The small circles represent the mean value for each participant. In B, solid lines indicate the linear regression lines. * indicates differences are statistically significant.

Supplementary Table 1

Slope (vel/mag)	Regression coefficient	SE	*t*	*d.f.*	*p*
'(Intercept)'	105.4189	1.7876	58.973	28216	0
'bkgd'	-0.3227	0.2246	-1.4365	28216	0.1509
'arousal'	0.0245	0.0602	0.4068	28216	0.6842
'valence'	0.0701	0.058	1.2089	28216	0.2267

Magnitude (deg)	Regression coefficient	SE	*t*	*d.f.*	*p*
'(Intercept)'	0.6565	0.0411	15.9887	28216	0.0000
'bkgd'	0.0386	0.0039	9.9198	28216	0.0000
'arousal'	0.0013	0.001	1.2599	28216	0.2077
'valence'	-8.76E-04	0.001	-0.871	28216	0.3838

Peak Velocity (deg/s)	Regression coefficient	SE	*t*	*d.f.*	*p*
'(Intercept)'	67.7853	4.3378	15.6268	28216	0.0000
'bkgd'	3.3471	0.3588	9.3287	28216	0.0000
'arousal'	0.1206	0.0961	1.2542	28216	0.2098
'valence'	0.0182	0.0927	0.1961	28216	0.8446

Supplementary Table 2

Slope (vel/mag)	Regression coefficient	SE	*t*	*d.f.*	*p*
'(Intercept)'	109.0271	4.1067	26.5486	2456	0.0000
'bkgd'	-1.7566	2.1639	-0.8118	2456	0.4170
'preparation'	-0.3022	2.3953	-0.1262	2456	0.8996
'bkgd:preparation'	0.1313	1.4194	0.0925	2456	0.9263

Magnitude (deg)	Regression coefficient	SE	*t*	*d.f.*	*p*
'(Intercept)'	0.6494	0.0722	8.9911	2456	0.0000
'bkgd'	0.0242	0.0357	0.6782	2456	0.4977
'preparation'	-0.0261	0.0395	-0.6608	2456	0.5088
'bkgd:preparation'	0.0086	0.0234	0.3681	2456	0.7128

Peak Velocity (deg/s)	Regression coefficient	SE	*t*	*d.f.*	*p*
'(Intercept)'	70.3268	7.1106	9.8904	2456	0.0000
'bkgd'	0.1493	3.415	0.0437	2456	0.9651
'preparation'	-2.7977	3.7791	-0.7403	2456	0.4592
'bkgd:preparation'	1.2172	2.2392	0.5436	2456	0.5868
